# Effectiveness of technology-assisted case management in low income adults with type 2 diabetes (TACM-DM): study protocol for a randomized controlled trial

**DOI:** 10.1186/1745-6215-12-231

**Published:** 2011-10-20

**Authors:** Leonard E Egede, Joni L Strom, Jyotika Fernandes, Rebecca G Knapp, Adebola Rojugbokan

**Affiliations:** 1 Center for Disease Prevention and Health Interventions for Diverse Populations, Ralph H. Johnson Veterans Affairs Medical Center, 109 Bee Street, Charleston, South Carolina, 29401 USA; 2 Department of Medicine, Division of General Internal Medicine & Geriatrics, Medical University of South Carolina, 135 Rutledge Avenue, Charleston, South Carolina, 29425 USA; 3 Center for Health Disparities Research, Medical University of South Carolina, Charleston, 135 Rutledge Avenue, Charleston, South Carolina, 29425 USA; 4 Department of Medicine, Division of Endocrinology, Diabetes, and Medical Genetics, Medical University of South Carolina, 96 Jonathan Lucas Street, Charleston, South Carolina, 29425 USA; 5 Department of Medicine, Division of Biostatistics and Epidemiology, Medical University of South Carolina, 135 Cannon Street, Charleston, South Carolina, 29425 USA; 6 Franklin C. Fetter Family Health Center, Inc., 51 Nassau Street, Charleston, South Carolina, 29403 USA

## Abstract

**Background:**

An estimated 1 in 3 American adults will have diabetes by the year 2050. Nationally, South Carolina ranks 10^th ^in cases of diagnosed diabetes compared to other states. In adults, type 2 diabetes (T2DM) accounts for approximately 90-95% of all diagnosed cases of diabetes. Clinically, provider and health system factors account for < 10% of the variance in major diabetes outcomes including hemoglobin A1c (HbA1c), lipid control, and resource use. Use of telemonitoring systems offer new opportunities to support patients with T2DM while waiting to be seen by their health care providers at actual office visits. A variety of interventions testing the efficacy of telemedicine interventions have been conducted, but the outcomes have yielded equivocal results, emphasizing the shortage of controlled, randomized trials in this area. This study provides a unique opportunity to address this gap in the literature by optimizing two strategies that have been shown to improve glycemic control, while simultaneously implementing clinical outcomes measures, using a sufficient sample size, and offering health care delivery to rural, underserved and low income communities with T2DM who are seen at Federally Qualified Health Centers (FQHCs) in coastal South Carolina.

**Methods:**

We describe a four-year prospective, randomized clinical trial, which will test the effectiveness of technology-assisted case management in low income rural adults with T2DM. Two-hundred (200) male and female participants, 18 years of age or older and with an HbA1c ≥ 8%, will be randomized into one of two groups: (1) an intervention arm employing the innovative FORA system coupled with nurse case management or (2) a usual care group. Participants will be followed for 6-months to ascertain the effect of the interventions on glycemic control. Our primary hypothesis is that among indigent, rural adult patients with T2DM treated in FQHC's, participants randomized to the technology-assisted case management intervention will have significantly greater reduction in HbA1c at 6 months of follow-up compared to usual care.

**Discussion:**

Results from this study will provide important insight into the effectiveness of technology-assisted case management intervention (TACM) for optimizing diabetes care in indigent, rural adult patients with T2DM treated in FQHC's.

**Trial Registration:**

National Institutes of Health Clinical Trials Registry (http://ClinicalTrials.gov identifier# NCT01373489

## Background

According to the Centers for Disease Control and Prevention, as many as 1 in 3 United States adults will have diabetes by 2050 [1]. As of 2007, approximately 23.6 million Americans or 7.8% of the population have diabetes; nearly 6 million of which have not been diagnosed [2]. In 2007, 1.6 million new cases of diabetes were diagnosed in individuals 20 years of age and older [2]. Currently, 10.7% of all people in this age group have diabetes [2]. In adults, type 2 diabetes accounts for about 90-95% of all diagnosed cases of diabetes [2].

South Carolina ranks 10^th ^in cases of diagnosed diabetes compared to other states [3]. The prevalence of diabetes in SC is presently 9.6%, with an estimated 300,000-350,000 people in SC living with diabetes. As observed nationally, more women and non-white individuals residing in SC are affected by diabetes [3]. Nearly 1089 deaths are attributed to diabetes, and 100,000-160,000 individuals are thought to be undiagnosed and affected by diabetes [3].

Diabetes is the leading cause of cardiovascular disease, strokes, blindness, and lower limb amputations and was the seventh leading cause of death listed on United States death certificates in 2006 [4] and in the 2009 Burden of Disease Report in South Carolina [3]. Diabetes is associated with significant morbidity, mortality, increased health care utilization, and increased health care costs [4]. The estimated economic cost of diabetes in the United States in 2007 was $174 billion with $116 billion spent directly in medical costs, and $58 billion used for indirect costs including lost workdays, disability, and premature mortality [2]. People diagnosed with diabetes have average medical expenditures that are approximately 2.3 times higher than what expenditures are for those not diagnosed with diabetes [2]. Additionally, the risk for death among people with diabetes is about twice that of people without diabetes of similar age [2].

Use of telephone contact, video-conferencing, personal digital assistants and web-based systems offer new opportunities to bridge the gap in support for patients with diabetes, between face to face visits with their health care providers; however, systematic reviews evaluating the efficacy of telemedicine interventions on patient outcomes have yielded equivocal results [5-7]. A consistent finding across all these reviews is that there is a shortage of controlled, randomized trials that implement clinical outcome measures using sample sizes with sufficient power. However, the nature of the interventions compared (telemonitoring, physician decision-making support, increased patient access to providers, electronic reminders, educational materials, or some combination of the above) and the baseline features of the populations studied have varied considerably, making specific conclusions difficult to draw.

While the optimal formula for technology-enhanced patient support is far from determined, there is a growing body of evidence to suggest that telemonitoring strategies, overall, are an effective intervention for improving metabolic control. Several small scale [8,9] and non-randomized [10,11] studies have found that patients exposed to telemonitoring interventions had lower Hemoglobin A1c (HbA1c) values than those without. Moreover, larger randomized controlled studies have also shown promising results. Two studies with Veteran populations demonstrated that home telemonitoring in conjunction with active care management resulted in lower HbA1c values at follow up when compared to standard care [12,13]. An open label study using ACCU-CHEK insulin guidance software also found that HbA1c values were significantly reduced in the experimental group and successfully maintained at one year follow-up [14]. In addition, adoption of a mobile (wireless) telemonitoring device with a diverse inner-city population was recently found to result in significantly reduced blood pressure and decreased blood sugar when compared to usual care [15].

But perhaps the most comprehensive randomized comparison of telemonitoring interventions yet completed is the IDEATel study with 1665 Medicare recipients [16]. Telemedicine case management was assessed in middle-aged Medicare recipients with diabetes living in federally designated medically underserved areas of New York State. Participants were randomized to one of two groups: usual care or intervention group. Those randomized to the intervention group received a home telemedicine unit capable of (1) videoconferencing with a nurse case manager, (2) remote monitoring of glucose and blood pressure with electronic upload and integration into the electronic medical records at the participating hospital, (3) dial-up Internet service allowing the patients to access their own clinical data and leave messages for the nurse case manager, and (4) access to an educational Website created by the American Diabetes Association (ADA). At 1 year, the mean HbA1c was significantly lower in participants in the intervention group, having improved from 7.35% to 6.97%. For those with a baseline HbA1c of 7 or greater, the HbA1c improved from 8.35% to 7.42%. Adjusted net reductions also favored the intervention group: HbA1c, 0.18% (p = 0.006), systolic and diastolic blood pressure, 3.4 mm Hg (p = 0.001) and 1.9 mm Hg (p < 0.001), and low density lipoprotein (LDL) cholesterol, 9.5 mg/dL (p < 0.001). Patients were also blindly assessed annually over a period of five years. It was found that the telemedicine group scored better than the standard care group on virtually all outcome measures at each annual evaluation.

In a smaller RCT of 30 patients with type 2 diabetes in Maryland [9], the feasibility of a cell phone-based diabetes software system in conjunction with an interactive web-based information management system were assessed. Compared to the control patients, those in the intervention group experienced decreases of 2.03% in their HbA1c levels (0.68% in the controls; *p *< 0.02, one tailed). In addition to improving diabetes outcome measures, 84% of those in the intervention group were able to facilitate optimal treatment decisions and have their medications changed or titrated (23%, *p *= 0.002).

Nurse case management has, in general, been shown to have a positive impact on treatment adherence, clinical outcomes and quality of life measures in chronic disease [17]. Evidence for the efficacy in the area of diabetes control is considerable. Several randomized controlled trials have shown that nurse case management can be an effective strategy for the reduction of HbA1c values in patients with diabetes [16,18-20]. Indeed, a recent meta-analysis of 29 studies found that nurse case management was a useful clinical strategy for the reduction of HbA1c values in patients with poorly controlled diabetes [21]. A recent study using a retrospective cohort design revealed that the use of nurse case management improved glycemic control with or without medication adjustment [22]. Even in studies in which HbA1c is not directly impacted, measures such as BP and emotional distress [23], and emergency department utilization [24] have improved.

**Figure 1:**
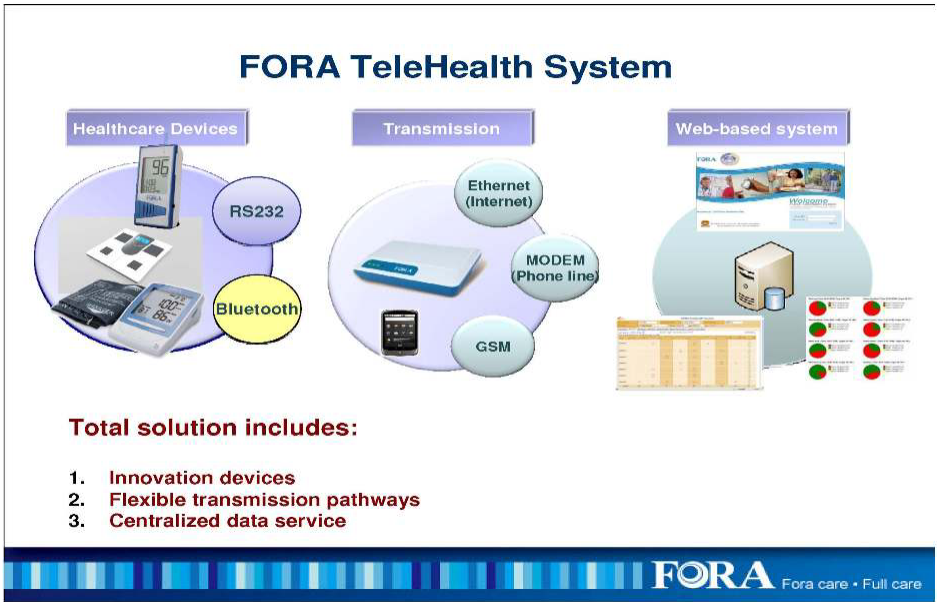
**The FORA Telehealth System**.

**Figure 2:**
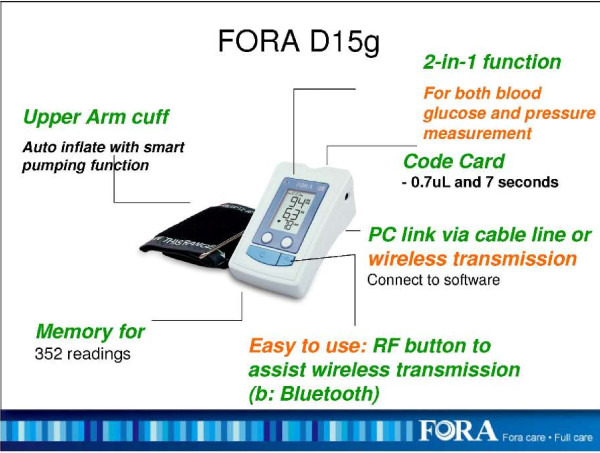
**The FOR A 2-in-1 Blood Glucose and Blood Pressure Machine (FORA D15g)**.

**Figure 3:**
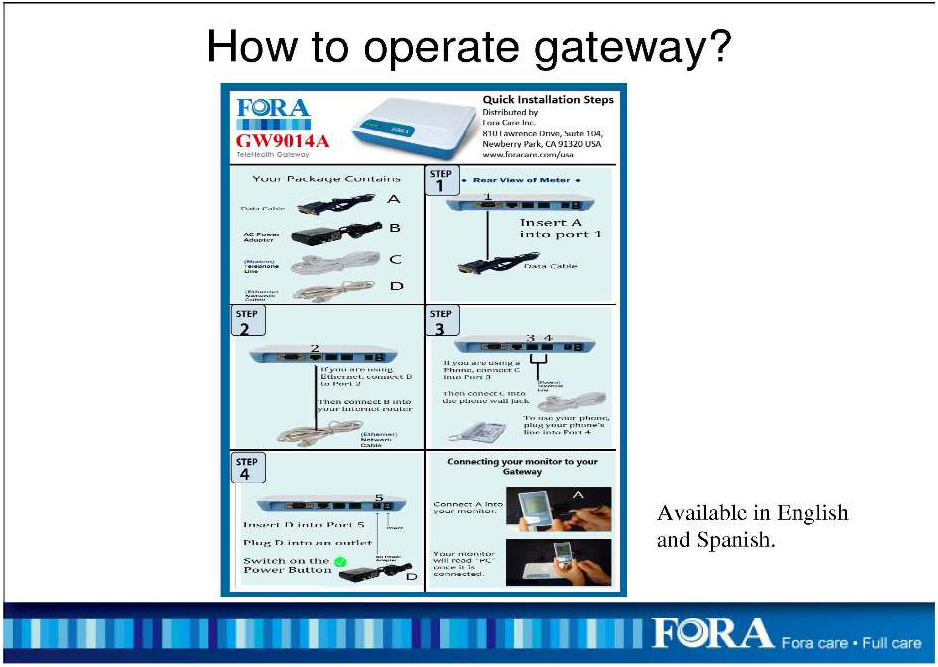
**Operation Instructions for the FORA Telephone/Ethernet Gateway**.

The FORA system is an inexpensive, off-the-shelf, state-of-the-art technology whereby a person/caregiver and a provider can communicate accurately on data needed for self-management of diabetes [25]. The system is comprised of a 2-in-1 Blood Glucose and Blood Pressure monitor (Figures 1 &2) which can provide an easy-to-use operation for users to accomplish two important tests. The measured results can be uploaded to a PC or web-based software by using a RS232 cable via a communication device to connect FORA glucose monitor and web-based FORA Telehealth System using a phone modem (Figure 3). The provider receives the readings, which are stored with previous readings for the patient. The provider can review the patient records on a secure website at any time. In addition, a nurse case manager can review the glucose and blood pressure readings daily and titrate medications as needed under the supervision of the patients' primary care provider. The widespread availability of analog phone lines presents an opportunity to employ a technology that uses a modem to transmit information via analog phone lines, to enhance patient-provider communication and adherence to treatment.

This paper describes the rationale, study aims and objectives, and research design and methods of an ongoing four-year, randomized clinical trial testing the effectiveness of technology-assisted case management intervention (TACM) using the FORA 2-in-1 and Telehealth System for diabetes in Federally Qualified Community Health Centers (FQHCs). The long-term goal of the project is to achieve improvement in diabetes-related outcomes in this patient population.

### Rationale

Numerous barriers to effective health care delivery in rural, underserved and minority communities have been identified. These include limited access to care, inability to afford medications, poor health literacy, and physician/provider mistrust. Conversely, studies have clearly shown that increased frequency of blood glucose testing, adherence to medications and more frequent provider visits dramatically improve glycemic control and outcomes in patients with diabetes. Sophisticated information technology, such as the FORA system, offers the potential to address several of the barriers to effective care by providing: (1) Reinforcement and positive feedback to patients that should increase adherence; (2) Real time access to blood glucose and blood pressure data for providers that should lead to more rapid optimization of treatment plans; and (3) Alerts to patients and providers if certain parameters are exceeded, e.g. sustained blood glucose levels above a pre-established threshold, triggering more rapid interventions. However to be successful, the technology needs to be implemented as part of an integrated diabetes management plan that is workable within a community health care setting. One can envision three levels of diabetes care, escalating in intensity: Usual Care: (Low Intensity) Typical office-based practice. In this setting the patient is custodian of his/her own care. Between scheduled visits, the patient is responsible for complying with prescribed treatment including medications, diet and exercise regimen, blood glucose monitoring, and follow up visits. Contact between scheduled visits is patient-initiated and occurs when the patient perceives a problem with the treatment plan. For patients with poorly controlled T2DM, which is often asymptomatic prior to the onset of serious complications, this is a particularly ineffective strategy. Case Management: (Intermediate Intensity). In this model, a clinic-based nurse case manager assumes some responsibility for patient compliance and chaperones them through the health care system. The intensity of the intervention may vary, but would typically include regular telephone contact with the patient between visits to reinforce compliance, provide reminders about scheduled appointments and inquire about glycemic control. The case manager notifies the physician when problems are identified. If the case manager is a qualified medical professional (e.g. a registered nurse or certified diabetes educator), then medication/dose changes may be implemented based on the phone contact according to a physician-approved protocol. The case management approach has previously been shown to be moderately effective in increasing patient adherence. In dealing with T2DM, the shortcomings are that contact is limited to prescheduled calls, the accuracy of data is limited by patient recall or recitation of blood glucose readings over the phone, and excepting the rare instances where the case manager has prescribing authority, changes require direct physician input and are implemented between scheduled visits only in cases where control is poor. Technology-Assisted Case Management (TACM): (High Intensity). This model capitalizes on information technology like the FORA system to link a case manager to poorly controlled diabetics in real time. Clearly a model that is applicable only to patients in need of intensive intervention, the advantages includes accurate data transfer to a medical decision maker and positive reinforcement/feedback to the patient via the FORA system to enhance adherence. If the case manager has physician-supervised prescriptive authority, then medication adjustments can be made daily or weekly, if needed, to achieve and maintain control. More frequent interventions, combined with improved compliance with medications and testing frequency, the strategies that are known to work, are enabled by this approach.

The study will use technology-assisted case management to target low income patients served in Federally Qualified Health Care Centers with poorly controlled T2DM residing in coastal South Carolina. Due to lack of access to care and low socioeconomic status, this population is at the highest risk for complications arising from uncontrolled T2DM.

### Study Aims & Objectives

The primary objective of this study is to test the effectiveness of technology-assisted case management intervention using the FORA 2-in-1 and Telehealth System for diabetes in improving HbA1c levels in indigent, rural adult patients with type 2 diabetes treated in Federally Qualified Community Health Centers. The primary outcome will be HbA1c at 6 months post-randomization, while the secondary outcomes will be blood pressure control and quality of life at 6 months post-randomization.

## Methods

The study is a 2 group randomized controlled trial with randomization of individual participants, blinded outcomes assessments at baseline, 3-months, and 6-months, and concurrent economic evaluation.

### Location and Setting

This study will be conducted primarily in eight community-based adult medicine primary care practices within the Franklin C. Fetter Family Health Center, Inc. Additional practices will be added if adequate sample is not obtained from the primary target practices. The Franklin C. Fetter Family Health Center, Inc. is a FQHC with eight adult medicine primary care clinics, 1 pediatric primary care clinic, a home health program, and two pharmacy locations. The clinics provide service to residents in Berkeley, Charleston, and Dorchester Counties of South Carolina. Demographic characteristics of the clinic population are as follows: 70% African American, 25% White, 5% Hispanic/other, 70% with income below 100% of federal poverty level, and 46% uninsured. The structure of each site is similar with the same number of practitioners and staff.

### Ethics and Trial Registration

The study is funded by grant W81XWH-10-2-0057 from the United States Department of Defense. The trial is approved by the Institutional Review Board (IRB) of the Medical University of South Carolina (Pro00009204). The trial is registered on the United States National Institutes of Health Clinical Trials Registry (http://ClinicalTrials.gov identifier# NCT01373489), available online at: http://clinicaltrials.gov/ct2/show/NCT01373489

### Trial Population and Recruitment

A total of 200 participants with T2DM will be randomized to one of two groups: 1) technology-assisted nurse case management or 2) usual care. We will use two complementary approaches to identify eligible study subjects. The first method will consist of systematic identification of patients with T2DM. We have obtained IRB approval for a partial waiver of HIPAA and will use clinic-billing records over the previous 12-month period to identify individuals with ICD-9 codes consistent with a diagnosis of type 2 diabetes. The physicians of eligible patients will be notified of their patients' potential eligibility and asked permission to enroll their patients in this study. After consent is obtained from the physicians, letters of invitation on clinic letterhead signed by the patient's physician will be mailed to patients from the study clinics. The letter will provide information about the study, explain the study requirements, and clarify that only individuals who meet certain criteria will be eligible to participate in the study. The letter will include an addressed and stamped post-card that individuals can mail back to indicate interest or lack of interest in participating in the study. In addition, the letter will provide a telephone number that interested individuals can call to receive detailed information about the study. In the letter, individuals will also be informed that they will receive a follow-up call in two weeks unless they mail back the post card or call to decline being contacted. Individuals who mail back the post card and express interest or call the provided telephone number will receive detailed information about the study. Individuals who agree to participate will be asked to provide written consent and will be scheduled for the initial screening assessment.

The second method will consist of referrals from physicians, other clinic staff such as nurses, or patients themselves in response to recruitment flyers for the study. The Principal Investigator will share the goals of the study and inclusion/exclusion criteria with physicians and clinic staff during clinic administrative meetings. Physicians and clinic staff will be asked to refer appropriate individuals to the study research assistants. In addition, IRB approved recruitment flyers will be posted in prominent locations in the study clinics.

Regardless of recruitment pathway, research staff members will obtain written informed consent, complete screening for eligibility, and assure that participants meet criteria for inclusion and participation in the study. The procedure and risks are explained to the patient and the consent form signed as per standard clinical practice. Individuals who meet eligibility criteria then complete the remainder of the assessment battery (see Tables 1).

**Table 1:** Data Collection Schedule

Questionnaires/Measurements	Baseline Visit	3-month visit	6-month visit
**Primary Outcome Measure**			

Hemoglobin A1c	X	X	X

			

**Secondary Outcome Measures**			

Blood Pressure	X	X	X

Quality of life (SF-12)	X	X	X

			

**Process Measures**			

Diabetes Knowledge Questionnaire	X	X	X

Diabetes Fatalism Scale	X	X	X

Perceived Diabetes Self Efficacy Scale	X	X	X

Summary of Diabetes Self-Care Activities Scale	X	X	X

Morisky Medication Adherence Scale	X	X	X

			

**Self-Report Measures**			

Patient Demographics	X		

Social support	X		

Health Literacy	X		

Depression (PHQ-9)	X		

Medical Comorbidity (Charlson Index)	X		

Service Delivery Perceptions/Treatment Credibility			X

Resource Use	X	X	X

### Randomization

All participants are randomly assigned to one of the two study arms (n = 100 per arm). The study coordinator will verify all eligibility criteria prior to randomization. The procedure and risks will be explained to the participants and the consent form signed as per standard clinical practice. All participants are randomly assigned to either the intervention (TACM) (n = 100) or usual care (UC) (n = 100) groups. Randomization takes place in waves, with approximately 50 subjects randomized every 6 months. The randomization sequence is generated by the research team biostatistician using a permuted block randomization scheme stratified by clinic site. The randomized assignment for eligible participants is accessible to the study coordinator using an envelope-based system at each clinic site. Once a randomization assignment is provided, the participant is entered into the study and will be included in the intention to treat analysis in accordance with the CONSORT guidelines [26].

### Intervention and Control Groups

There is one active treatment group (technology-assisted case management) and a usual care group.

#### Overview of the Technology-assisted Case Management (TACM) Intervention

The TACM intervention uses the FORA 2-in-1 Telehealth System for diabetes to link a case manager to poorly controlled patients with diabetes in real time. Participants will be assigned the FORA 2-in-1 Telehealth System and provided glucose test strips to allow testing at least once a day. They will be asked to perform glucose testing and blood pressure measurement using the FORA system once daily. They will be asked to upload the measurements daily as soon as possible after the test is performed. The nurse case manager will have access to a secure server to which the uploaded measurements are stored in real time. Under the supervision of the patient's primary care provider, the nurse case manager will make medication adjustments weekly based on the uploaded readings and an evidence-based treatment algorithm approved by the primary care provider. The TACM intervention in essence facilitates increased frequency of self-monitoring and titration of medications in response to test values to optimize diabetes and blood pressure control. Details of the TACM intervention are provided below.

#### Algorithms for Glucose and Blood Pressure Control

We developed two separate algorithms for control of hyperglycemia and high blood pressure based on American Diabetes Association and American Association of Clinical Endocrinologists guidelines in collaboration with physicians at the study clinics. The algorithms went through an iterative process to ensure that evidence was tailored to the unique needs of FQHCs. For example we chose generic versions and low cost alternatives when possible. We also tried to use medications that were on formulary at the respective study clinics. The nurse case manager will be trained to follow the approved algorithms, but the ultimate decision for medication titration will rest with the treating physician.

#### Glycemic and Blood Pressure Control Targets

The following indices will be used to determine whether participants are meeting treatment goals. These goals conform to current standard of care in outpatient diabetes management.

• Fasting/pre-prandial Blood Glucose:          < 110 mg/dl (range 70-109 mg/dl)

• Bedtime Blood Glucose:                            < 130 mg/dl (range 70-129 mg/dl)

• Two-hour post-prandial Blood Glucose:    < 140 mg/dl (range 110-139 mg/dl)

• HgbA1C target:                                        < 7.0%

• Blood pressure                                        < 130/80 mmHg

#### Role of the Case Manager

The case manager is to serve as an adjunct to usual care by providing more frequent contact between the patient and his/her health care provider. This will be accomplished through: (1) Real time tracking of patient-generated blood glucose and blood pressure data using the FORA 2-in-1 and Telehealth System for diabetes; (2) Minimum of monthly phone calls to participants to reinforce diabetes self-management behaviors, e.g. medication compliance, blood glucose monitoring, diet/exercise programs; (3) More frequent phone calls as needed for patients undergoing active changes in management; (4) Telephone reminders about upcoming scheduled appointments; and (5) Use of uploaded blood glucose and blood pressure data and information obtained from participant contact to determine need for additional interventions. The case manager will function as liaison between the primary provider and the participant, identifying those participants in need of additional intervention and facilitating implementation of a revised treatment plan.

#### Case Manager Telephone Follow-up

The nurse case manager will be responsible for conducting regular follow-up by telephone with all TACM intervention participants. The initial phone follow-up will occur within two weeks of the initial study visit. Thereafter, the minimum frequency of phone follow-up will be monthly for participants who are meeting treatment targets based upon weekly review of uploaded data. More frequent contact is permitted for participants not meeting treatment targets and in whom active interventions are ongoing. Routine follow-up calls will review the following: (1) Interval health-related events and changes in medications; (2) Current level of glycemic and blood pressure control, based on data logged in the FORA system; (3) Frequency and severity of hypoglycemia on the current therapy based on both FORA system data and patient perception of hypoglycemia; (4) Reinforce adherence with diet/exercise program and medications; (5) Reinforce participant education relating to caring for diabetes, treatment of hyper/hypoglycemia and safe driving rules; (6) Remind participants about upcoming scheduled appointments with their primary provider and/or study visits; and (7) Answer participants' questions.

#### Usual Care Group

Apart from study visits, participants will be followed by their Franklin C. Fetter PCPs. The provider will be responsible for determining treatment parameters, making changes in the treatment regimen, and determining the timing of follow up visits. Between scheduled office encounters, contact between participant and provider will be patient initiated. The provider may use clinic nurses to follow up on problematic patients or patients with abnormal results. In essence, this group will receive the current standard of care at the study clinics.

### Study Instruments and Data Collection Schedule

**Figure 4:**
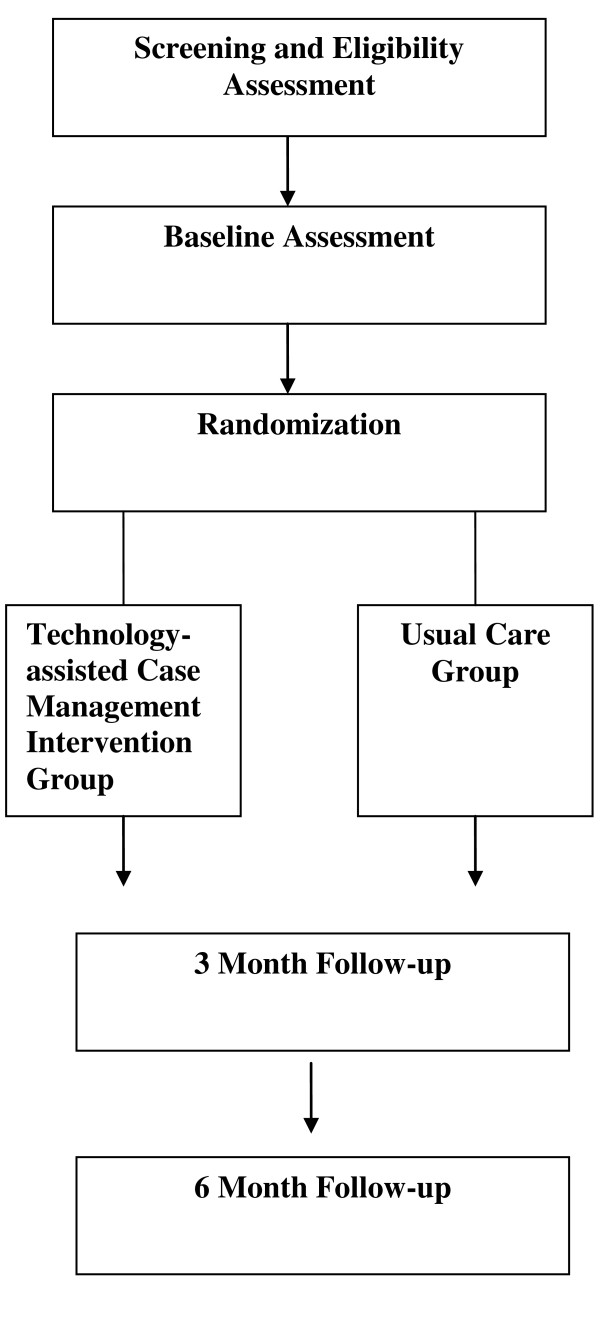
**Description of Study Design and Study Flow**.

See Figure 4 and Tables 1 and 2, for the study design and study flow, data collection schedule, and data collection measures and instruments, respectively.

**Table 2:** Data Collection Measures and Instruments

Measure	Data Collected	Method
**Primary Outcome**	Hemoglobin A1c	Blood specimens will be obtained at baseline, 3-, and 6-months visits.

**Secondary Outcomes**	Blood Pressure	Blood pressure readings will be obtained at baseline, 3-, and 6-months visits.

	Quality of life	Quality of life will be measured by the SF-12 [31], which is a valid and reliable instrument to measure functional status.

		

**Process Measures**	Information	This will be measured by the 24-item Diabetes Knowledge Questionnaire (DKQ) [32]

	Motivation	This will be measured with the 12-item Diabetes Fatalism Scales (DFS) [33].

	Self-Efficacy	This will be measured by the perceived diabetes self-management scale (PDSMS) [34].

	Behavioral Skills	This will be assessed with the Summary of Diabetes Self-Care Activities (SDSCA) scale [35].

	Medication Adherence	This will be measured with the new 8-item self-report Morisky Medication Adherence Scale (MMAS) [36].

		

**Self-report measures**	Demographics	Previously validated items from the 2002 National Health Interview Survey [37] will be used to capture age, gender, race/ethnicity, marital status, household income, and health insurance.

	Social support	The Medical Outcomes Study (MOS) Social Support Survey [38] will be used to measure social support.

	Health Literacy	The abbreviated version of the Test of Functional Health Literacy in Adults (S-TOFHLA) [39] is designed to rapidly screen patients for potential health literacy problems.

	Depression	The PHQ-9 is a brief questionnaire that scores each of the 9 DSM-IV criteria for depression [40].

	Medical Comorbidity	The patient's history of medical comorbidity will be documented using a standardized and validated questionnaire [41].

	Service Delivery Perceptions	This will be assessed with 5 items that have been previously validated in mental health studies. The items were slightly modified to be relevant to diabetes.

	Treatment Credibility	To assess for differences in outcome expectancy, a modified treatment credibility scale developed by Borkovec and Nau (1972) will be used [42].

	Resource Utilization & Cost	The perspective of cost will be that of the payer. Previously validated questions on resource utilization will be administered as a part of the baseline, 3-, and 6-month visits.

### Intervention Delivery, Treatment Integrity, and Treatment Adherence

A full-time registered nurse (RN) will conduct the screening, patient scheduling, consent, and enrollment procedures as well as deliver the case management intervention. A blinded research assistant (RA) will conduct all study assessments. A data coordinator will enter all study related data into a secure web-based data system and perform all related data tracking and coordination duties.

#### Training and Supervision of Interventionist (Nurse Case Manager)

Training in the basic elements of the treatment algorithms and ongoing supervision and oversight of the case manager will be provided by the study physicians (a board certified endocrinologist and a board certified internist). Training will consist of two full days of didactic training on diabetes and hypertension in year 01 and then one day booster sessions in years 2-5 to maintain knowledge. In addition, the case manager will receive two full days of training on the FORA system and use of the secure website in Yr 01 and a full day of booster training in subsequent years. The nurse case manager will also participate in weekly research meetings to discuss challenging patients and resolve conflicts. The study physicians will be available by telephone at all other times to support the nurse case manager.

#### Treatment fidelity checks

In order to ensure that treatments are competently administered in accordance with the study protocol, a 20% random sample of intervention study charts will be reviewed along with the uploads from the secure site for the corresponding patients to ensure the case manager is adhering to the treatment algorithms. To evaluate adherence, rating forms will be developed based upon the treatment protocol to determine if the nurse case manager escalated treatment as appropriate and how well she accomplishes a range of relevant tasks for each patient. The study physicians will review and rate the randomly selected patient charts to assess for fidelity to treatment.

#### Patient adherence to treatment protocol

This is a very important aspect of this study given the intensity of the intervention protocol. We will adopt the following strategies to ensure an optimal level of compliance: (1) At enrollment, we will stress the importance of daily testing with the FORA system and daily upload of data; (2) We will place reminder telephone calls to participants on a weekly basis to remind them to test and upload data; (3) As described earlier, we will request the names and telephone numbers of three of the participant's friends and/or relatives who know how to reach the participant in the event we are unable to reach him or her; (4) The research staff will be flexible in accommodating participants' schedules and every attempt will be made to schedule research visits to coincide with clinic appointments. In addition, we will reimburse the participants ($25 each) for baseline, 3-, and 6-month follow-up visits.

#### Treatment enactment

The behaviors that are the target of this intervention are daily testing and uploading of blood glucose and blood pressure results from the FORA system. We will assess adherence to these behaviors based on review of frequency of uploads to the secure server.

### Primary Outcome Measure

The primary outcome is HbA1c level at 6 months of follow-up.

### Sample Size Determination and Power Analysis

For the continuous longitudinal clinical and quality of life outcomes for Primary Aims 1 and 2 (e.g., trajectory of HbA1C, BP, and SF-12 total scores), with 80 participants randomized 1:1 to each intervention group (TACM or UC), we will have 85% power to detect at least a 0.4 standardized (sd units) effect size between the TACM vs. UC intervention groups [assuming 3 time points (baseline, 3 and 6 months); intra-class correlation no greater than 0.6; level of significance (α) = 0.05, two-tailed ]. To account for dilution effect of ITT analyses and "administrative" dropout, i.e., those who drop out immediately upon randomization (do not receive a treatment or return for a post baseline visit), we inflated the sample size by 20% to achieve a final ITT sample size of 100 subjects randomized to each intervention group (N = 200).

### Data Analysis

#### Preliminary Descriptive Analyses

Baseline values for demographic, clinical, and other putative prognostic variables will be compared for imbalance across the two treatment conditions (TACM; UC). These analyses will identify potential confounding variables to be used as covariates in subsequent analyses and will include race/ethnicity, gender, age, social support, health literacy, concurrent medical illness, depression, motivation etc. To investigate potential limits on generalizability, we will compare characteristics of the premature exits/protocol non-adherent with those who were completers/protocol adherent. Characteristics of participants who do not complete the study or do not comply with the treatment will be compared for both conditions.

#### Statistical Analysis Plan

The longitudinal trajectory of primary and secondary clinical (HbA1C, blood pressure) and quality of life outcomes will be compared using the generalized linear mixed models (GLMM) approach. In addition to accommodating a wide range of distributional assumptions [dichotomous/categorical (e.g., binomial), continuous (e.g., normal), ordinal, count (e.g., Poisson)], the approach accommodates missing data, time varying or invariant covariates, and multilevel data (clustering) such as repeated measurements on participants and possible cluster effects within clinics through inclusion of random effects in the model [27]. For the continuous outcomes such as blood pressure and HbA1C, GLMM is equivalent to a linear mixed effect model (MEM) approach. MEM analyses estimate individual change in outcome for each participant in addition to estimating average change in outcome within each intervention arm. Covariables, such as age, race, gender, medical comorbidity, social support, health literacy, and depression severity, will be added to the MEM model to adjust for putative confounding variables. For the dichotomous outcome, proportion (%) of participants who dropout prior to the 6-months end-of-study time point, we will compare proportion dropout for the TACM vs. UC intervention groups using GLMM for a binary outcome [equivalent to logistic regression analysis for the single end-of-study endpoint]. Appropriate contrasts will be specified to obtain individual time point comparisons of interest (e.g., 3 months, 6 months). Variables will be considered as fixed (e.g., primary intervention effect) or random (e.g., clinic) as appropriate.

##### Exploratory analyses

In additional exploratory analyses, we will examine the effect modification of baseline factors, such as race/ethnicity age, gender, social support, health literacy, medical comorbidity) on the relationship between clinical/quality of life outcomes and intervention using GLMM/MEM analyses as described above. These analyses involve the inclusion of a putative moderator variable (e.g. race/ethnicity) by intervention interaction term in the multivariable model. A significant interaction effect would suggest that the effect of the putative moderator on the outcome of the intervention is different across levels of the moderator variable. For example, a significant race × intervention interaction would suggest that one intervention (e.g., TACM) may be more beneficial for African Americans than Whites.

We will also explore the potential mediating effect of changes in diabetes knowledge, motivation, self-efficacy, and behavioral skills on intervention effectiveness outcomes using the GLMM modeling approach. These analyses address the question of whether the putative mediating variables help explain the relationship between the effectiveness outcomes (e.g. improvement in HbA1C or blood pressure control) and the primary independent variable (intervention group). It is posited that variation in intervention accounts for variation in the mediators (e.g. changes in diabetes self-care activities) and variations in mediators account for variations in intervention effectiveness (improvement in HbA1C or blood pressure control). For these analyses, we will follow the recommended methods as described by Baron & Kenney, 1986; Holmbeck, 1997; and MacKinnon et al., 2007 [28-30], and conclusions will be carefully considered in light of the assumptions that accompany mediation analysis.

## Discussion

The study was funded in June 2010. Our Institutional Review Board (IRB) and second level administrative review by the Department of Defense IRB were obtained in August 2011. Study recruitment date is anticipated to start in October 2011, with all follow-up assessments associated with the study expected to be completed by July 2014.

The study is inherently novel in that it optimizes two strategies that have been shown to improve glycemic control: nurse case management and telemonitoring interventions to improve effective health care delivery in rural, underserved and minority communities with type 2 diabetes. The study provides a practical and sustainable system of diabetes management that will help low income patients achieve and maintain goals within established treatment guidelines regardless of geographic location. The study employs novel and innovative information technology to improve communication between patients and providers and improve patient adherence with prescribed therapy. It is expected that the results of this study would enhance the efficiency of care, speed the implementation of effective treatment plans, generate sustained improvement in glycemic control, decrease the morbidity and early mortality associated with T2DM, and ultimately generate significant reductions in overall health care costs.

## Abbreviations

BP: Blood Pressure; CONSORT: Consolidated Standards of Reporting Trials; FQHC: Federally Qualified Health Center; GLMM: Generalized Linear Mixed Models; HbA1c: Hemoglobin A1c; HIPAA: Health Insurance Portability and Accountability Act; ICD-9: The International Classification of Diseases; 9^th ^revision; IRB: Institutional Review Board; LDL: Low Density Lipoprotein; MEM: Mixed Effect Model; PI: Principal Investigator; PCP: Primary Care Physician; RA: Research Assistant; RCT: Randomized Control Trial; SF-12: The 12-Item Short Form Health Survey; T2DM: Type 2 Diabetes Mellitus; TACM: Technology-assisted Case Management; UC: Usual Care

## Competing interests

The authors declare that they have no competing interests.

## Authors' contributions

LEE conceived of the study; LEE obtained funding for the study. LEE, JF, and RGK participated in the design and coordination of the study. LEE, JF, RGK, and AR reviewed the manuscript. JLS and LEE drafted and finalized the manuscript. All authors read and approved the final manuscript.
